# Factors involved in the development of the metabolic **syndrome**. We are what we eat and what we are eating

**DOI:** 10.1186/1687-9856-2013-S1-O3

**Published:** 2013-10-03

**Authors:** Hiroaki Masuzaki

**Affiliations:** 1 Division of Endocrinology, Diabetes and Metabolism, Hematology, and Rheumatology, Second Department of Medicine, Graduate School of Medicine, University of the Ryukyus, Okinawa, Japan

## 

A variety of metabolic and molecular changes in brain and adipose tissue play a critical role in the pathophysiology of life style-related metabolic diseases [1-3]. Even in obese subjects with insulin resistance in skeletal muscle and liver, insulin action on adipose tissue remains intact or rather exaggerated, resulting in considerable difficulties in weight reduction. Excess in circulating insulin thus causes body fat gain as well as ectopic lipid overload in liver, skeletal and cardiac muscle, pancreas and vasculature. The adipocyte hormone, leptin controls feeding behavior, augments fatty acid β-oxidation in the skeletal muscle and enhances whole body insulin sensitivity, thereby serving as a promising therapeutic candidate for the treatment of obesity-diabetes syndrome. However, the clinical application of leptin has been hampered by the notion that leptin does not fully exert its metabolic effects in subjects with fat diet-induced obesity [4-6]. It is important to note that the future risk of cardiometabolic diseases exists for infants with low birth weight/intrauterine growth retardation (IUGR). IUGR causes a premature leptin surge in the neonatal period in mice, and conceivably, through a number of epigenetic mechanisms, it may induce hypothalamic leptin resistance in adulthood. Mechanisms of non-genomic, intergenerational transmission of metabolic diseases from parents to children have also been unveiled in an expeditious fashion. The endoplasmic reticulum (ER) is an intracellular organelle involved in protein folding and apoptosis. The accumulation of misfolded proteins in the ER, termed as ER stress, is involved in the molecular pathophysiology of type2 diabetes and metabolic syndrome. Our recent research in mice demonstrated that high fat diet-induced ER stress in the hypothalamus plays a pivotal role in the preference for fatty foods and resultant increase in body weight. We provided the first evidence that brown rice and its major component, γ-oryzanol, ameliorate glucose dyshomeostasis in mice fed high-fat diet (HFD), accompanied by reduction of hypothalamic endoplasmic reticulum (ER) stress [7]. In my talk, I try to review the update of mechanisms of metabolic syndrome, with a particular focus on the molecular food sciences.
